# Technology Enabled Remote Monitoring in Schools(TERMS): A Case Study Series Using Parallel Testing in Clinical Settings and School Workshops

**DOI:** 10.1192/bjo.2024.239

**Published:** 2024-08-01

**Authors:** Hafeesa Sameem, Alka Ahuja, Gemma Johns, Mrs Vicky Simmons, Oliver John

**Affiliations:** 1 Aneurin Bevan University Health Board, Newport, United Kingdom; 2 RCPsych Wales, Cardiff, United Kingdom

## Abstract

**Aims:**

The TERMS (Technology Enabled Remote Monitoring in Schools) project aimed to elucidate the operational dynamics of remote monitoring with bluetooth-enabled physical health monitoring devices. The focus was on measuring key parameters such as usage, perceived value, accuracy, and satisfaction among patients, their families, and healthcare staff. Additionally, we sought to explore the potential future integration of remote monitoring in educational settings through school site workshops.

**Background:**

Digital healthcare has become an indispensable part of effective healthcare provision on a global level. Remote monitoring is the use of technology, to monitor patients outside of a clinical setting with the help of medical devices, questionnaires, and clinical dashboards, allowing clinicians to review the data to assist in clinical assessment and decision-making. While this method is already established for conditions like Diabetes and Asthma it is not for other conditions like ADHD. This is especially a challenge for the younger demographic.

Schools are pivotal for promoting student well-being and early interventions, leading to reduced negative outcomes like exclusion and school absence and enhanced academic attainment. The TERMS project strives to bridge the gap between education and healthcare by collaborating with schools and clinicians. This is in alignment with the digital and data strategy for health and social care in Wales as outlined by the Welsh Government(2023).

**Methods:**

This study had 2 parts:

**Clinical Site Testing:**

Blue tooth-enabled clinical monitoring device readings were obtained after they were monitored first with traditional clinical monitoring devices. Additional qualitative feedback was also obtained.

**Educational Workshops:**

Workshops were carried out with students and teaching staff to collect qualitative and quantitative feedback on the remote monitoring equipment and patient-facing dashboard. This also set out to determine if remote monitoring in schools is feasible and how it could be implemented.

**Results:**

A total of 47 clinical patient cases were included. The accuracy of the bluetooth-enabled device readings and those of traditional equipment were compared. Analysis of the qualitative data revealed useful domains and subdomains of opinions along with the user-friendliness of the software interface.

**Conclusion:**

Overall, we have identified that patient and family perception of remote monitoring is positive, suggesting an improved/comparable level of care for their condition. Additionally, school workshops highlight that this service could be implemented within a school setting. As long as considerations were made for who would conduct the remote monitoring and what the role of the school would be.

